# Visualization of childhood allergic diseases based on VOSviewer and CiteSpace

**DOI:** 10.3389/fmed.2025.1615154

**Published:** 2026-01-08

**Authors:** Yan Wang, Xiaoyu Liu, Wenze Cui, Yinghua Hu, Wenhuan Song, Likun Zhu, Zujun Wang, Xiaolu Ji, Youpeng Wang

**Affiliations:** 1 Heilongjiang University of Chinese Medicine, Harbin, China; 2 Weihai Central Hospital, Weihai, China; 3 Binzhou Medical College Affiliated Traditional Chinese Medicine Hospital, Binzhou, China; 4 Binzhou Medical University, Yantai, Shandong, China; 5 Binzhou People's Hospital, Shandong, China; 6 Second Affiliated Hospital of Heilongjiang University of Chinese Medicine, Harbin, China

**Keywords:** eczema, allergic rhinitis, bronchial asthma, cough-variant asthma, bibliometric analysis, childhood

## Abstract

**Introduction:**

Childhood allergic diseases, such as eczema, allergic rhinitis, bronchial asthma, and cough-variant asthma, are a growing health concern around the world. There is a lot of research about these diseases, but a clear and complete study is still needed to better understand them and help guide future research.

**Methods:**

This study used a bibliometric analysis of research on childhood allergic diseases from 2014 to 2024. The main goal was to find patterns in publications, main researchers, research focuses, teamwork between groups, and new topics. Data were collected from Web of Science, Scopus, and PubMed. The study included English-language articles and reviews only. The tools VOSviewer and CiteSpace were used to study publication patterns, where research was done, which authors and journals worked together, how often papers were cited together, which papers were cited the most, and how keywords appeared and formed groups.

**Results:**

The amount of research was different for each disease. Eczema got the most attention and kept growing. Cough-variant asthma had fewer studies. The United States and China were the main countries that did most of the work and had well-known authors. The focus of studies changed from general studies about disease spread to more detailed topics like the microbiome, genetics, special treatments, environmental causes, other health problems that happen together, and the effects of COVID-19. The way researchers worked together was not the same for all diseases. This showed that research was more developed and better connected for some diseases and less developed for others, mainly for cough-variant asthma.

**Conclusion:**

This study gives a short look at recent research on childhood allergic diseases. It shows that eczema research is growing fast, but cough-variant asthma is still studied much less, with only 110 papers in 10 years. This big difference shows a clear lack of knowledge. These results can help make better research plans and improve medical care in this field.

## 1 Introduction

### 1.1 The growing global health challenge of childhood allergic diseases

This study examines four major childhood allergic diseases: eczema (atopic dermatitis) (1), allergic rhinitis (2), bronchial asthma (3), and cough-variant asthma (4). These diseases are a big problem in child healthcare and public health. They were chosen because they are key parts of what is called the “allergic march.” This means they involve the skin (eczema), the upper airways (allergic rhinitis), and the lower airways (bronchial asthma and cough-variant asthma). These are some of the most common long-term illnesses in children. They share similar disease processes, so studying them together helps in understanding allergic problems in the whole body. Other diseases like food allergy are also important, but they have different immune causes and treatment methods, so they were not included. This makes the study focused on skin and airway allergic diseases.

Such diseases are prevalent worldwide, with incidence rates either high or increasing, and impact the immediate and long-term health of millions of children. Eczema, the most common chronic inflammatory skin disease in young children, is one that causes intense itching and seriously decreases the quality of life of affected families. It is often also regarded as the initial stage of this person's “allergic process” (5), and is one of the key examples for understanding the range of childhood allergen diseases, so that the focus of this research is to shed light on them. Allergic rhinitis is also the most common chronic lower airway disease in children and is associated with recurrent nasal symptoms, which adversely interrupt sleep and learning. It can also be comorbid with asthma (6). It can be regarded as one of the important associations in studying the relationship between the upper and lower airway diseases. Bronchial asthma, or the most common chronic lower airway disease in children (7), is another chronic allergic disease and is characterized by recurrent airway inflammation and hyper-responsiveness. In addition, this disease poses a substantial burden on the health of young children, posing a significant threat to their lung health in the long term; hence, it is of great importance in this study. Both cough-variant asthma, characterized by chronic coughing, is often misdiagnosed because of its abnormal presentation. It stands out as less discussed and understudied, although it is consistent with airway inflammation, which may evolve or transform into typical asthma (8). Meanwhile, these disorders all interfere with the body's normal physiological functions for healthy function, including sleep, breathing, and activity, implying that they significantly affect psychological development, socializing, learning ability, and overall quality of life for children (9). The chronic nature, co-morbidities, and socio-economic costs of these illnesses call for meticulous and systematic research. This study is essential for advancing Pediatrics, and addressing rapidly developing global health challenges to ensure the well-being of children, thereby emphasizing that a detailed understanding of current trends, hotspots, and research frontiers in this domain is critical for optimizing clinical practice and guiding future research pursuits.

### 1.2 The need for a bibliometric analysis of allergic diseases in children

With a drastic rise in clinical and social interest, the body of scientific research literature in pediatric allergic diseases has grown rapidly during the past decade. The plethora of research papers, reviews, and reports reflects vigorous advancement within the domain, but it is also challenging researchers to comprehend the overall knowledge structure, crucial advances, and future trends in time-satisfying ways. Traditional methods of literature review struggle to cope with such massive body of information. As per this requirement, bibliometrics, an objective and quantitative method of analysis, is especially needed (10). With the help of Bibliometrics, it is simple to systematically organize huge volumes of literature data, depict macro-trends, hot points, major contributors, and collaboration networks in the form of visual maps, providing a structured overview of the entire knowledge (11). This study employs two leading bibliometric software tools, VOSviewer and CiteSpace, leveraging their complementary strengths. VOS viewer is able to generate clear and static-network maps of collaborations and co-occurrences while Cite Space excels in temporal analysis, detecting emerging trends and pivotal points in the timeline of research. We combine these two programs to yield a comprehensive and dynamic overview. This paper lays the scientific framework of our study, whereby we have used bibliometric techniques to analyze the literature on major pediatric allergic diseases, the objective being to present the current status and trends of the domain objectively and to provide valuable references for future studies and clinical practice.

## 2 Research methodology

### 2.1 Data sources and search strategies

Data were collected from three significant academic databases to inform the current research study. The databases included were the Web of Science Core Collection (WoSCC), Scopus, and PubMed. Each search was terminated on 1st April 2025 to ensure the findings of our study extracted the latest published data between 2014 and 2024. To ensure a broad representation of the pediatric allergic diseases, our literature search targeted four major disorders: Eczema (Atopic Dermatitis), Allergic Rhinitis, Bronchial Asthma, and Cough-Variant Asthma. Methods of the search strategy are described in the Annex as well.

The search strategy was conducted using a combination of subject terms (TS), title-abstract-keywords (TITLE-ABS-KEY), and medical subject headings (MeSH Terms). This combo of terms was utilized to achieve precision in searches. The search was confined to literature related to Children and encompassed keywords such as “children”, “infantile”, “child”, “childhood”, and “pediatric” to get the search as per the Children's context. Further, it focused on literature by the English language and targeted literature categorized under “Article” and “Review”. Literature like editorial materials, Meeting Abstracts, Letters, News Items, Book Chapters, etc., which do not pertain to academic research, was excluded to keep the research-centric results.

### 2.2 Data collection and processing

Separately, two scientists performed a literature search to maximize completeness. Selection of the articles was done according to the Preferred Reporting Items for Systematic Reviews and Meta-Analyses (PRISMA), which is illustrated in the PRISMA flow chart (Annex B for a flow diagram). Following the search, records of articles, including their titles, authors, abstracts, keywords, journals, years of publication, references, citation data, authors' affiliation details, country/region details, were extracted in plaintext form from all the three database entries and saved in EndNote (ris) format (12).

Since the data structure, field names (such as author, country, institution) and the naming conventions of the four databases vary in different ways, directly merging them might cause the results to be biased. Accordingly, raw data from the three databases were stored and combined using EndNote X9, and detailed checking helped to remove the duplicate records. The final dataset contained 18,734 records belonging to eczema, 10,262 to allergic rhinitis, 14,937 to bronchial asthma, and, 110 to cough-variant asthma after deduplicating and combining. The merged repository was used as the raw dataset for the following bibliometric analysis.

### 2.3 Bibliometric analysis and visualization

**Figure 1:**
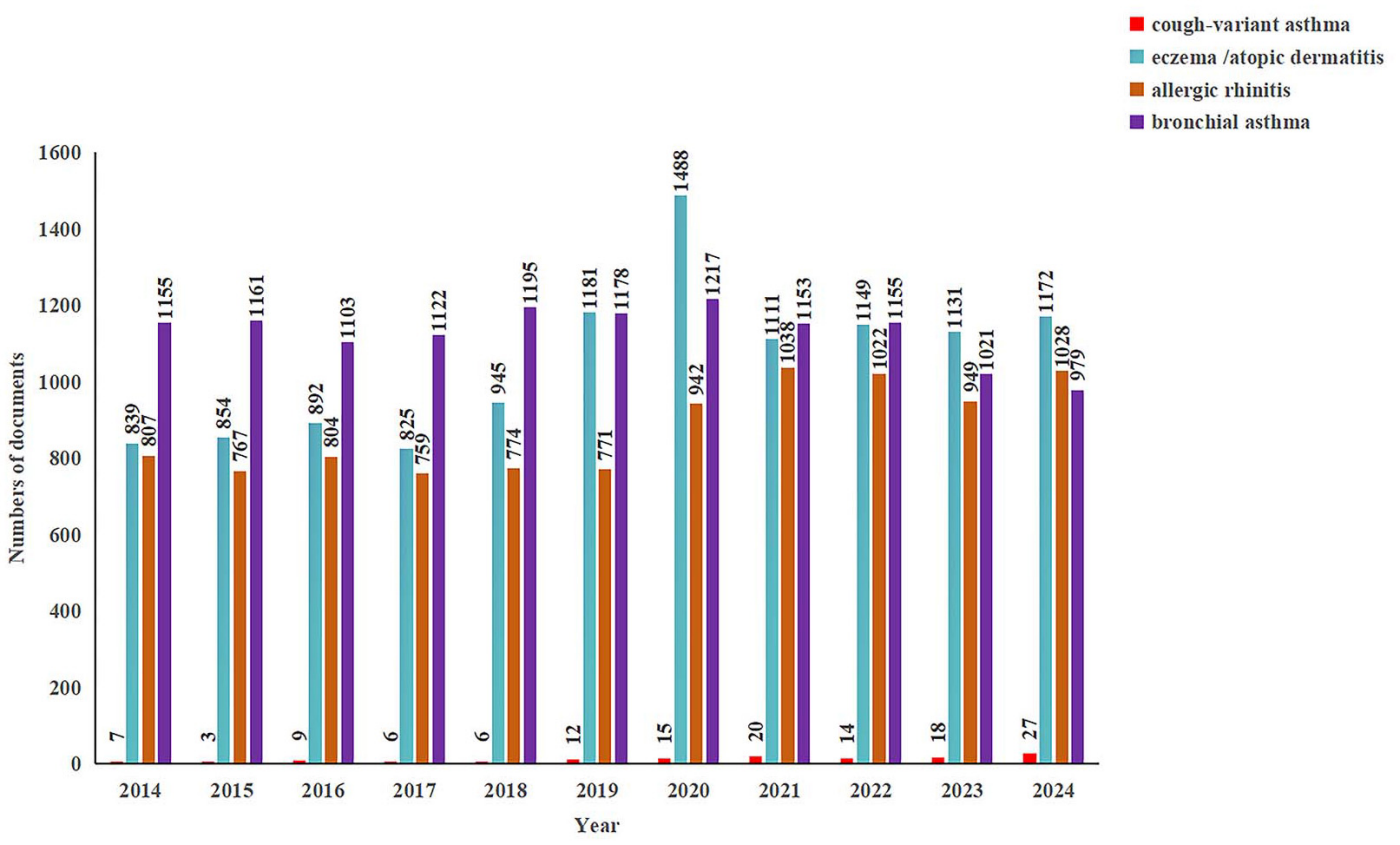
Bibliometric analysis of eczema, bronchial asthma, allergic rhinitis. Cough-variant asthma (2014–2024).

**Figure 2:**
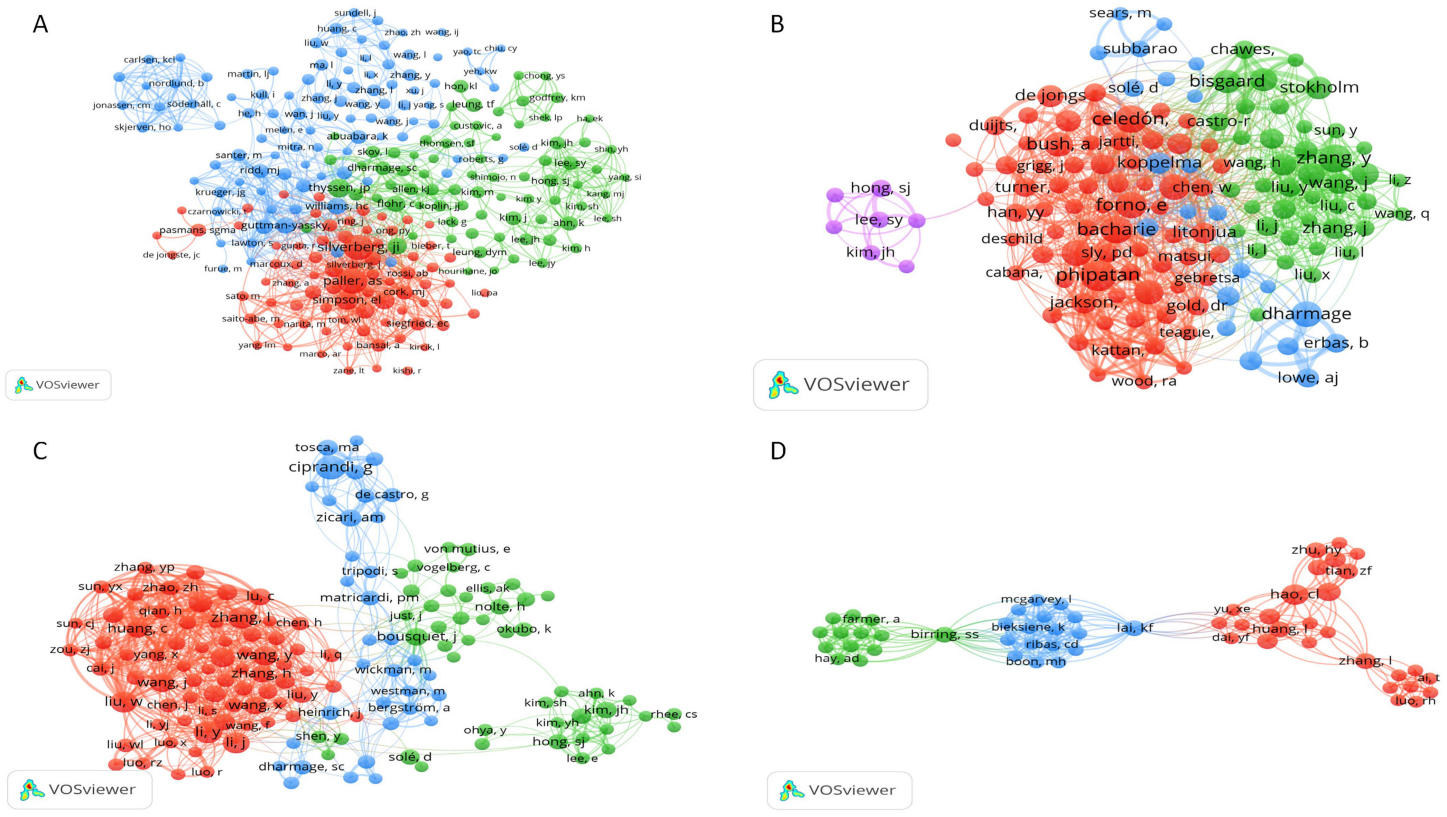
Bibliographic coupling networks of authors. **(A)** Bibliographic coupling network for eczema; **(B)** Bibliographic coupling network for bronchial asthma; **(C)** Bibliographic coupling network for allergic rhinitis; **(D)** Bibliographic coupling network forcough-variant asthma. In these networks, each node represents an author. Node size corresponds to the number of publications. Lines between nodes indicate that two authors cite a common third work, with thicker lines denoting a stronger relationship. Node color represents distinct author clusters.

**Figure 3:**
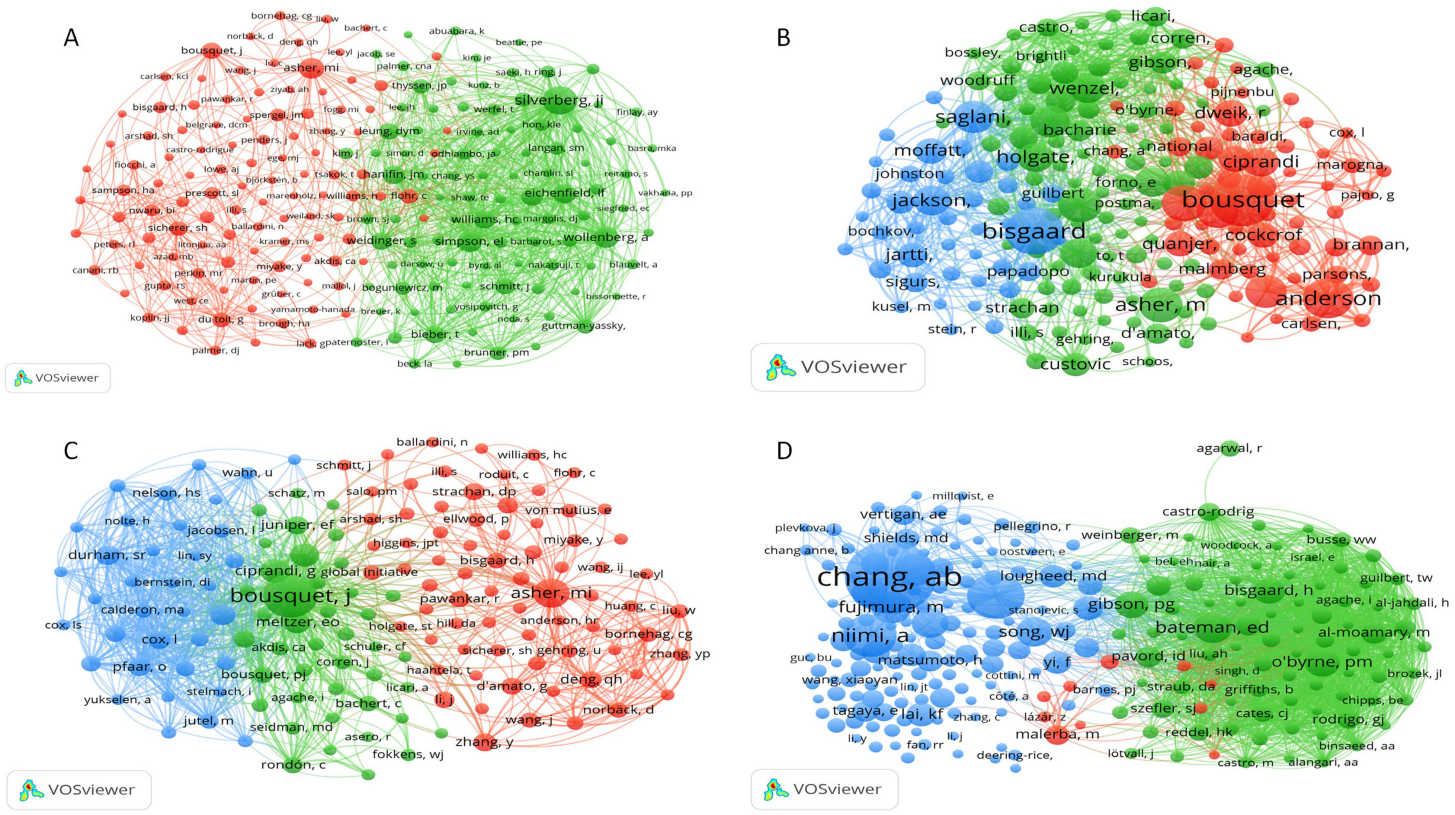
Co-citation networks of authors. **(A)** Co-citation network for eczema; **(B)** Co-citation network for bronchial asthma; **(C)** Co-citation network for allergic rhinitis; **(D)** Co-citation network for cough-variant asthma. In these networks, each node represents an author. Node size is proportional to the number of times the author has been co-cited. Lines between nodes indicate a co-citation relationship, with thicker lines showing higher frequency. Node color represents distinct author clusters.

The principal tools employed in this study were bibliometric data management and visualization software VOSviewer (VOSviewer Version 1.6.20) and CiteSpace (CiteSpace Version 6.4. R1). We chose these tools for two reasons: VOSviewer is particularly effective for visualizing static structural networks (e.g., co-authorship, keyword co-occurrence), while CiteSpace delivers excellent results in temporal analyses, such as detecting keyword bursts and constructing timeline views, offering a more dynamic examination of how the research landscape developed. Microsoft Excel 2021 was also used to organize basic data and graph annual publication trends. The analysis included several specific elements:

(1) Publication pattern analysis: we used Excel to count the publication quantities by year for each disease domain and constructed a line figure (Figure 1) emphasizing research trends from 2014 to 2024.
(2) Volume analysis of countries/regions and research institutions. VOS viewer and Cite Space considered publication volumes, cooperation networks, and countries/regions, and research institutions' centrality. Our analysis found that those countries/regions and institutions had the most publications (Table 1) and constructed collaboration network figures.
(3) Author and co-cited author analysis:
  Author analysis: we examined the core author groups and their authors' cooperation networks via VOS viewer (Figure 2). We counted both highly producing authors, publication amounts, mean publication years, and the corresponding citation quantities (Table 2).
  Co-Citation authors analysis: we identified influential scholars in each domain and their citation counts via VOS viewer or Cite Space by constructing co-cited author net (Figure 2), with the results indicating the base of the research domain and knowledge structure.
(4) Analysis of journals and co-cited journals:
  Journal analysis: we collected statistics about major journals publishing the relevant literature, their publication counts, and combined this with impact factors (IF) and JCR partition information (Table 3), which were used to evaluate the journal influence in the field co-cited journals. We used VOSviewer or CiteSpace to construct the co-citation network among these journals with co-cited authors to detect relevant core sources and their citation frequency (Table 3).
(5) Analysis of highly cited and co-cited literature:
  Highly cited literature review: in each domain of disease, we identified literature with the best citation counts (Table 4). We then analyzed the type of themes and contributions, revealing key research results in this domain.
  Co-cited literature review: CiteSpace conducted co-citation cluster analysis (Figure 5) and emergent literature detection to identify frontiers of research and milestone the important works (Figure 6).
(6) Keyword analysis:
  Keyword co-occurrence analysis: VOS viewer constructed a coefficient co- occurrence network figure (Figure 7), revealing hotspots in research.
  Keyword Clustering and Timeline Diagram: CiteSpace clustered and analyzed keywords, generated cluster labels, and drew a keyword timeline view (Figure 8) to show the research topic's evolutionary path.
  Keyword Emergence Analysis: CiteSpace detected keywords with emergent growth (Figure 8) to identify research frontiers and trends.

**Table 1:** Top 10 countries/ regions and organizations related to eczema, allergic rhinitis, bronchial asthma, and cough-variant asthma.

**Type**	**Rank**	**Country**	**Documents**	**Rank**	**Organization**	**Documents**
Eczema	1	USA	4,656	1	University of Copenhagen	440
	2	China	3,543	2	Harvard Medical School	425
	3	Japan	1,776	3	University of California	410
	4	Italy	1,563	4	Karolinska Institute	366
	5	South Korea	1,465	5	Northwestern University Feinberg School of Medicine	365
	6	UK	1,400	6	Imperial College London	355
	7	Germany	1,250	7	University of Toronto	336
	8	France	929	8	Icahn School of Medicine At Mount Sinai	322
	9	Australia	924	9	University of Southampton	306
	10	Turkey	814	10	Capital Medical University	267
Allergic Rhinitis	1	China	5,267	1	Karolinska Institutet	325
	2	USA	3,626	2	Capital Medical University	263
	3	Italy	1,843	3	Humboldt University of Berlin	238
	4	South Korea	1,676	4	Free University of Berlin	236
	5	Germany	1,429	5	China Medical University Taiwan	234
	6	Spain	1,190	6	Harvard University	225
	7	UK	1,135	7	Institut National De La Sante Et De La Recherche Medicale (Inserm)	223
	8	Japan	1,062	8	Berlin Institute of Health	220
	9	France	1,009	9	National University of Singapore	212
	10	Sweden	807	10	Chang Gung Memorial Hospital	210
Bronchial Asthma	1	USA	12,787	1	Harvard Medical School	1,000
	2	China	8,392	2	University of California	850
	3	Australia	4,019	3	University of Copenhagen	801
	4	Canada	2,679	4	Karolinska Institutet	516
	5	Netherlands	2,677	5	Cincinnati Children's Hospital Medical Center	508
	6	Italy	2,463	6	Imperial College London	501
	7	Spain	2,430	7	Boston Children's Hospital	381
	8	Germany	2,364	8	University Medical Center Rotterdam	344
	9	France	2,129	9	Icahn School of Medicine At Mount Sinai	341
	10	Japan	2,017	10	University of Toronto	337
Cough-Variant Asthma	1	China	199	1	Nanjing University of Chinese Medicine	9
	2	USA	35	2	Sun Yat Sen University	8
	3	UK	19	3	Tabriz University of Medical Sciences	8
	4	Saudi Arabia	17	4	King Faisal Specialist Hospital And Research Center	7
	5	Japan	13	5	Erasmus Mc	6
	6	Italy	9	6	Erasmus University Rotterdam	6
	7	Netherlands	9	7	King Saud University	6
	8	Canada	8	8	Queens University—Canada	6
	9	Australia	6	9	Shengjing Hospital of China Medical University	6
	10	France	6	10	Capital Institute of Pediatrics (CIP)	5

**Table 2:** Top 10 authors and co-cited authors related to eczema, allergic rhinitis, bronchial asthma, and cough-variant asthma.

**Type**	**Rank**	**Author**	**Avg. pub. year**	**Documents**	**Citations**	**Rank**	**Co-cited author**	**Citations**
Eczema	1	Silverberg, JI	2020	229	3,058	1	Silverberg, JI	1,294
	2	Paller, AS	2020	223	5,119	2	Wollenberg, A	653
	3	Simpson, EL	2020	126	5,115	3	Asher, MI	637
	4	Thyssen, JP	2020	118	1,064	4	SIMPSON, EL	617
	5	Eichenfield, LF	2020	101	3,444	5	Eichenfield, LF	558
	6	Guttman-Yassky, E	2020	90	2,245	6	Hanifin, JM	506
	7	Irvine, AD	2020	88	1,429	7	Paller, AS	461
	8	Williams, HC	2019	87	3,927	8	Weidinger, S	445
	9	Flohr, C	2020	86	1,225	9	Williams, HC	433
	10	Ohya, y	2020	85	869	10	Bieber, T	424
Allergic Rhinitis	1	Ciprandi, G	2020	78	438	1	Bousquet, J	1,277
	2	Li, Y	2019	67	242	2	Asher, MI	528
	3	Zhang, L	2020	63	630	3	Ciprandi, G	338
	4	Liu, W	2019	61	864	4	Brozek, JL	331
	5	Li, J	2020	60	573	5	Meltzer, EO	278
	6	Wang, Y	2020	49	186	6	Canonica, GW	243
	7	Zhang, Y	2020	49	357	7	Durham, SR	215
	8	Wang, J	2019	46	609	8	Zhang, Y	194
	9	Zhang, X	2021	46	656	9	Cox, L	192
	10	Huang, C	2019	42	972	10	Pfaar, O	188
Bronchial Asthma	1	Phipatanakul, W	2020	134	172	1	Bousquet, J	256
	2	Zhang, YY	2020	115	72	2	Bisgaard, H	226
	3	Celedón, JC	2020	110	75	3	Anderson, SD	182
	4	Bacharier, LB	2020	109	145	4	Martinez, FD	153
	5	Forno, E	2019	102	51	5	Asher, MI	150
	6	Szefler, SJ	2018	97	112	6	Miller, MR	147
	7	Weiss, ST	2019	97	531	7	Crapo, RO	140
	8	Dharmage, SC	2019	96	1,060	8	Saglani, S	137
	9	Bush, A	2019	91	3,314	9	Busse, WW	132
	10	Gern, JE	2019	88	459	10	Sears, MR	122
Cough-Variant Asthma	1	Hao, CL	2019	4	36	1	Chang, AB	58
	2	Yu, XM	2019	3	28	2	Morice, AH	30
	3	Birring, SS	2017	2	525	3	Niimi, A	27
	4	Huang, L	2019	2	17	4	Fujimura, M	18
	5	Jiang, WJ	2019	2	17	5	Irwin, RS	18
	6	Lai, KF	2020	2	490	6	Bateman, ED	17
	7	Tian, ZF	2020	2	19	7	Corrao, WM	16
	8	Wang, YQ	2019	2	17	8	Dicpinigaitis, PV	16
	9	Yuan, YF	2020	2	19	9	Song, WJ	16
	10	Zhang, L	2021	2	11	10	O'Byrne, PM	15

**Table 3:** Top 5 journals and co-cited journals related to eczema, allergic rhinitis, bronchial asthma, and cough-variant asthma.

**Type**	**Rank**	**Journal**	**Documents**	**IF**	**JCR**	**Rank**	**Co-cited journal**	**Citations**	**IF**	**JCR**
Eczema	1	Allergy	388	12.6	Q1	1	J Allergy Clin Immune	16,902	11.4	Q1
	2	Journal of Allergy and Clinical Immunology	235	11.4	Q4	2	Allergy	7195	12.6	Q1
	3	British Journal of Dermatology	217	11	Q1	3	Brit J Dermatol	6,171	11	Q1
	4	Pediatr Allergy Immunol	189	4.3	Q2	4	Clin Exp Allergy	4,416	6.3	Q2
	5	Pediatr Dermatol	189	1.2	Q4	5	J Am Acad Dermatol	4,128	12.8	Q1
Allergic rhinitis	1	Allergy	243	12.6	Q1	1	J Allergy Clin Immun	12,324	11.4	Q1
	2	Pediatr Allergy Immunol	119	4.3	Q2	2	Allergy	8,132	12.6	Q1
	3	Journal of Allergy and Clinical Immunology	109	8.2	Q1	3	Clin Exp Allergy	4,026	6.3	Q2
	4	Pediatric Allergy and Immunology	92	4.3	Q2	4	Ann Allerg Asthma Im	2,780	5.8	Q2
	5	Allergol Immunopathol (MADR)	77	2.5	Q4	5	Pediat Allerg Imm-UK	2,739	4.3	Q2
Bronchial asthma	1	J Asthma	775	1.7	Q4	1	J Allergy Clin Immun	7,058	11.4	Q1
	2	Pediatr Pulmonol	471	2.7	Q3	2	Am J Resp Crit Care	4,808	19.3	Q1
	3	J Allergy Clin Immunol	450	11.4	Q1	3	Eur Respir J	3,502	16.6	Q1
	4	Pediatr Allergy Immunol	392	4.3	Q2	4	Allergy	2,619	12.6	Q1
	5	J Allergy Clin Immunol Pract	380	8.2	Q1	5	Thorax	2,092	9	Q1
Cough-variant asthma	1	Medicine	6	1.3	Q4	1	Chest	191	9.5	Q1
	2	Journal of Asthma	3	1.7	Q4	2	J Allergy Clin Immun	171	11.4	Q1
	3	Journal of Biological Regulators and Homeostatic Agents	3	0.8	Q4	3	Am J Resp Crit Care	169	19.3	Q1
	4	Pediatric Pulmonology	3	2.7	Q3	4	Eur Respir J	161	16.6	Q1
	5	Allergology International	2	6.2	Q2	5	Thorax	111	9	Q1

**Table 4:** Top 5 references related to eczema, allergic rhinitis, bronchial asthma, and cough-variant asthma.

**Type**	**Rank**	**Literature**	**Title**	**Doi**	**Source**	**IF/JCR**	**Citations**
Eczema	1	Eichenfield (2014b)	Guidelines of care for the management of atopic dermatitis section 2. Management and treatment of atopic dermatitis with topical therapies (28)	doi: 10.1016/j.jaad.2014.03.023	Journal of the American Academy of Dermatology	12.8/Q1	926
	2	Tamburini (2016)	The microbiome in early life: implications for health outcomes (29)	doi: 10.1038/nm.4142	Nature Medicine	58.7/Q1	782
	3	Jakobsson (2014)	Decreased gut microbiota diversity, delayed bacteroidetes colonization and reduced th1 responses in infants delivered by cesarean section (30)	doi: 10.1136/gutjnl-2012-303249	Gut	23/Q1	690
	4	Sidbury (2014)	Guidelines of care for the management of atopic dermatitis (31)	doi: 10.1016/j.jaad.2014.03.030	Journal of the American Academy of Dermatology	12.8/Q1	672
	5	Asher (2014)	Global burden of asthma among children (32)	doi: 10.5588/ijtld.14.0170	International Journal of Tuberculosis and Lung Disease	3.4/Q3	470
Allergic rhinitis	1	Roberts (2018)	European position paper on rhinosinusitis and nasal polyps 2020 (33)	doi: 10.4193/rhin20.600	Rhinology	4.8/Q2	1,754
	2	Huang (2019d)	Atopic dermatitis (34)	doi: 10.1016/s0140-6736(15)00149-x	Lancet	98.4/Q1	1,409
	3	D'amato (2015)	Eaaci guidelines on allergen immunotherapy: allergic rhinoconjunctivitis (35)	doi: 10.1111/all.13317	Allergy	12.6/Q1	511
	4	Calderón (2015b)	Prevalence, risk factors, and management of asthma in china: a national cross-sectional study (36)	doi: 10.1016/s0140-6736(19)31147-x	Lancet	98.4/Q1	425
	5	Simpson (2020)	Meteorological conditions, climate change, new emerging factors, and asthma and related allergic disorders. A statement of the world allergy organization (37)	doi: 10.1186/s40413-015-0073-0	World Allergy Organization Journal	3.9/Q2	371
Bronchial asthma	1	Chung (2014)	International ers/ats guidelines on definition, evaluation and treatment of severe asthma (38)	doi: 10.1183/09031936.00202013	European Respiratory Journal	16.6/Q1	2,864
	2	Soriano (2017)	Global, regional, and national deaths, prevalence, disability-adjusted life years, and years lived with disability for chronic obstructive pulmonary disease and asthma2015 (39)	doi: 10.1016/s2213-2600(17)30293-x	Lancet Respiratory Medicine	38.7/Q1	1,693
	3	Papi (2018)	Asthma (40)	doi: 10.1016/s0140-6736(17)33311-1	Lancet	98.4/Q1	1,480
	4	Fahy (2015)	Type 2 inflammation in asthma—present in most, absent in many (41)	doi: 10.1038/nri3786	Nature Reviews Immunology	27.7/Q2	1,165
	5	Dharmage (2019)	Epidemiology of asthma in children and adults (42)	doi: 10.3389/fped.2019.00246	Frontiers in Pediatrics	7.1/Q1	679
Cough-variant asthma	1	Morice (2020)	Ers guidelines on the diagnosis and treatment of chronic cough in adults and children (43)	doi: 10.1183/13993003.01136-2019	European Respiratory Journal	16.6/Q1	482
	2	Guc (2014)	The assessment and management of chronic cough in children according to the British thoracic society guidelines: descriptive, prospective, clinical trial (44)	doi: 10.1111/crj.12076	Clinical Respiratory Journal	1.9/Q4	35
	3	Sun (2019)	Montelukast and budesonide for childhood cough variant asthma (45)	doi: 10.29271/jcpsp.2019.04.345	JCPSP-Journal of the College of Physicians and Surgeons Pakistan	0.7/Q4	18
	4	Wei (2019)	Clinical efficacy of montelukast sodium combined with budesonide or combined with loratadine in treating children with cough variant asthma and influence on inflammatory factors in the serum (4)	doi: 10.3892/etm.2019.7574	Experimental and Therapeutic Medicine	2.4/Q4	18
	5	Uryasjev (2020)	The cough variant asthma (46)	doi: 10.26442/00403660.2020.03.000404	Terapevtiche skii Arkhiv	0.3/Q4	17

In the visual network graphs created by VOSviewer and CiteSpace, nodes represent analysis objects (e.g., countries, institutions, authors, keywords), and node sizes reflect their publication numbers, citations, or occurrence frequency. Lines between nodes indicate cooperative, co-occurring, or co-cited relationships, and their thickness represents relationship strength. Different colors distinguish clusters or time periods.

## 3 Results

### 3.1 Trends in the number of publications

After harmonizing and consolidating the data, the final dataset included 18,734 articles on eczema, 10,262 on allergic rhinitis, 14,937 on bronchial asthma, and 110 on cough-variant asthma. Figure 1 shows the publication trends for these four childhood allergic diseases from 2014 to 2024. Eczema was the topic studied the most, producing a growing number of papers from 2014 to 2024, with the number of studies reaching 1,544 in 2019 and peaking at 1,488 in 2020. Therefore, the steady increase between 2019 and 2024 implies that eczema has become an increasingly important topic of research, particularly within the context of allergic conditions in pediatric populations. in addition to the prevalence of eczema and its clinical burden, as well as the potential impact on the young people's lives (13). Based on this analysis, it seems reasonable to assume that the research interest in this topic will continue to grow in the foreseeable future.

Bronchial asthma ranks second in terms of publication volume. As it can be seen in Figure 1, from 2014 to 2023, the number of publications stayed consistent with an average of about 1,100, yet we also observe a noticeable drop in print volumes beginning in the year 2022, which dropped to 979 by the year 2024. While interest is clearly still high in research in this area, this trend suggests a possible maturity of the research fields or a repositioning of research emphasis that does not continue the broad studies on bronchial asthma, as contrasted with the growth of eczema research. The fluctuations of these print volumes over time could be indicative of variations in research interests, availability of resources, or other aspects influencing research in this field.

Allergic rhinitis, among child allergic conditions, is one of the overlooked conditions in comparison to eczema and bronchial asthma. Posters on allergic rhinitis reported fairly steady numbers from 2014 to 2019 and after an inconclusive rise between 2020 and 2021, this rise lasted poorly for one year only, then, over the next three-year period, publications on allergic rhinitis declined, and stabilized once again in 2019 with a tiny number at 1,028 publications by 2024, demonstrating the widespread international agreement and long-sought continued, albeit fluctuating, interests of researcher(s)s in studying allergic rhinitis in children, with the prevalence of allergic rhinitis being probably one reason for this continuation of research (14).

Conversely, cough-variant asthma has the poorest research interest among the four diseases. In 2014, only seven relevant publications were released, whereas fluctuations resulted in a maximum of 27 studies by 2024. This trend indicates that though there is a clinical indication for research studies of cough-variant asthma, international attention is scarce, and no relevant studies were found. While cough-variant asthma (15) takes primary attention in the guidelines issued by GINA, the lack of knowledge and the imperfect distribution of research resources probably resulted in the insufficient number of studies done.

### 3.2 Distribution of countries and institutions

Having analyzed the research distribution by countries and institutions, a few discrepancies in the number of studies were found as a consequence of the different methods of combining the data. These differences were however quite negligible, and the general trend remained similar. It can be concluded that, during the last 10 years, research was performed globally in many countries and institutions about childhood allergic diseases; only in the case of imbalance.

According to Table 1, the research on eczema and bronchial asthma in the United States is the highest, with four thousand six hundred fifty-six (4,656) and 12,787 (the highest globally) articles, respectively. However, research of allergic rhinitis and cough-variant asthma in China takes up first spot with 5,267 and 199 articles, respectively. Notably, Japan, Italy, and South Korea have made significant contributions to pediatric allergic disease research.

In China, Capital Medical University, China Medical University Taiwan, Nanjing University of Chinese Medicine, and Sun Yat-sens University have all produced a large handful of highly cited publications. For an look at the Us, Harvard Medical School, the University of California, and Cincinnati Children's Hospital Medical Center have made essential contributions to research into eczema and bronchial asthma. At a high level, childhood allergic disease is a field where the United States and China dominate.

### 3.3 Distribution of authors and cited authors

A network analysis of the children's allergic disease cohort study (Figure 2) demonstrates consistent research patterns and interaction networks for different conditions. E-maw (Figure 2A), which has received the highest attention, has the most intricate research networks. The blue network with the center at Silverberg, JI, was special and depicts a wide concern for research around the condition and a growing cross-disciplinary research trend.

Bronchial asthma (Figure 2B), with less literature, displays a powerful network composed of Phipatanakun, W, and Zhang, Y. Multiplethed clusters denote loose global collaboration, suggesting a well-established centralized architecture has failed to form, despite advancements in pharmacological (16), epidemiological (17), and environmental studies (18).

Allergic rhinitis (Figure 2C). There's a somewhat dominant but loosely-related trend, with a few teams focusing on Ciprandi, G, who've made major contributions to certain areas of research. Cough variation asthma (CVA) (Figure 2D), which is relatively underdeveloped at the moment, also displays a notable decline with publications. There's a subset of dozens of authors, led byHUOAOCHaoCL, who's made notable research contributions in the course of years, but its teamSize remains scant and loosely related. There are several potential bottlenecks for how this area could develop in the future.

Regarding core scholars and thematic focal points, the analysis of co-cited authors (Figure 3; Table 2) provides some perspective. The field of eczema investigation has been largely researched but is still quite dispersed. The most frequently cited scholar is Silverberg, JI, with 229 publications and 3,058 references. Asher, MI, and Wollenberg, A make substantial contributions to mechanistic (19) and epidemiologic studies (20), respectively.

An active and centralized network in bronchial asthma (Figure 3B). Bousquet, J has 1277 citations and leads research in co-morbitbidities. Eastern of them are Bisgaard, H, who specializes in intestinal flora (21); Asher, MI an expert in epidemiological research; and Ciprandi, G has 78 publications and 438 citations; the condensed network means high potential for collaboration in this field.

Allergic rhinitis (Figure 3C) has a relatively loose network, dominated by Bousquet, J, with 256 citations. Key contributions are from Asher, MI, focusing on epidemiology; Durham, SR, addressing immunotherapies (22); and Phipatanakul, W, with 134 articles and 172 citations. Research has delved into big data and therapeutics for clinical translation.

CVA (Figure 3D) is in early, fragmented stages. Chang, AB, and Bateman, ED, are key experts and etiologic cores. Hao, CL, with 4 articles and 36 citations, has limited impact, but research is expected to deepen as attention increases.

### 3.4 Distribution of journals and co-cited journals

**Figure 4:**
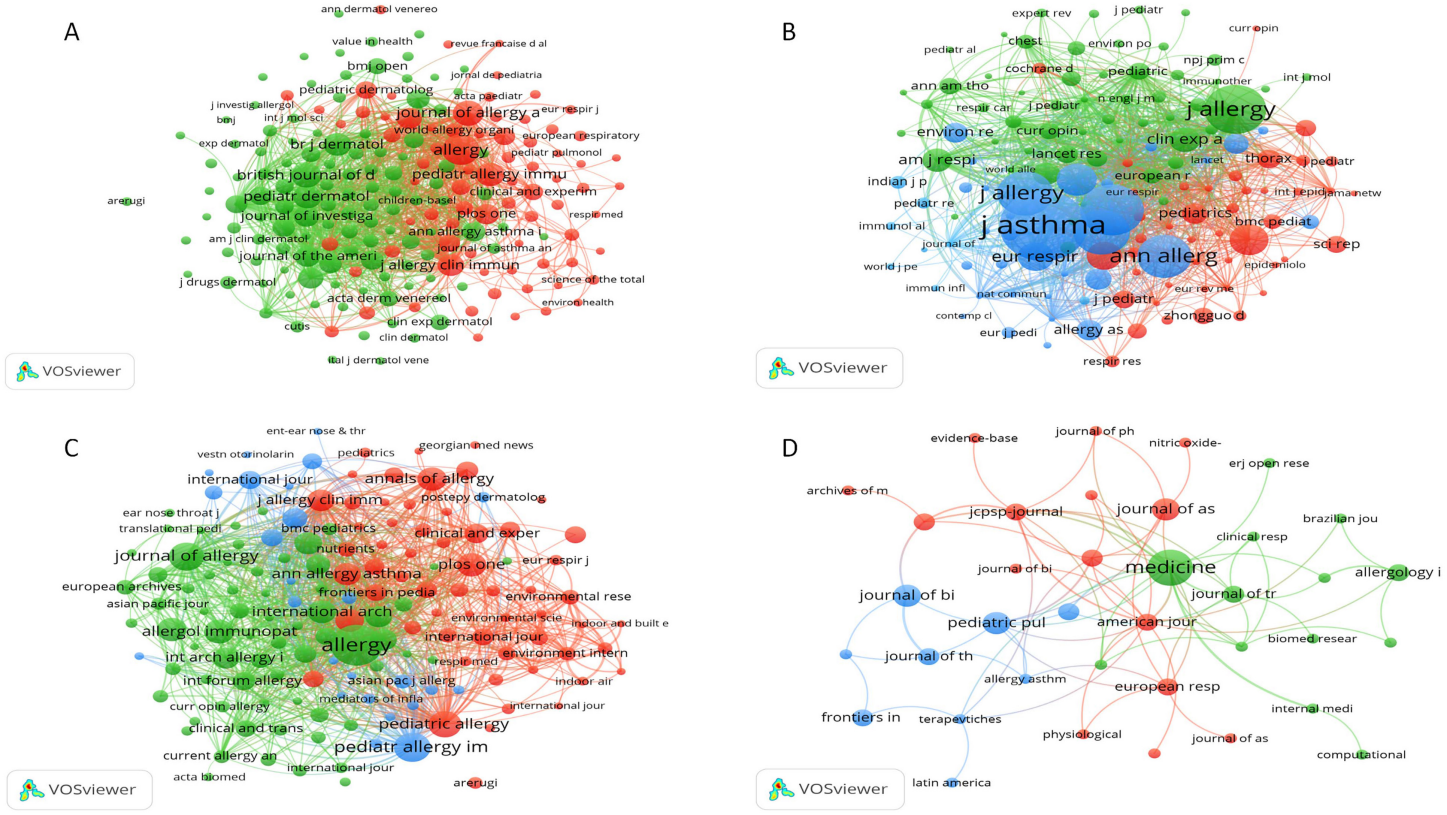
Bibliographic coupling networks of journals. **(A)** Bibliographic coupling network for eczema; **(B)** Bibliographic coupling network for bronchial asthma; **(C)** Bibliographic coupling network forallergic rhinitis; **(D)** Bibliographic coupling network for cough-variant asthma. In these networks, each node represents a country or region. Node size is proportional to the number of publications from that country. Lines between nodes indicate a co-authorship relationship, with thicker lines representing stronger collaboration. Node color represents distinct country clusters.

Table 3 and Figure 4 examine the citation patterns found in academic journals and identify the key research hotspots, hotspot trends, and the contribution of each journal toward childhood allergic disease research. The interrelationship between literature sources demonstrates the key roles played by eczema, bronchial asthma, allergic rhinitis, and cough variant asthma and the major role played by some cornerstone journals.

A centralized academic network has evolved in eczema research, and among all journals reviewed, a few are leading in the study of childhood allergic diseases. “Allergy” and “Journal of Allergy and Clinical Immunology” are the two most cited and should thus be perceived as the key dissemination channels. Studies largely tend to concentrate on the pathophysiology (23) and treatment of eczema, notably the biomedical effectiveness of several medications (24).

Over ten years, “Allergy” published 388 publications on eczema, and its impact factor was 12.6, ranking in the first quartile (Q1) of Journal Citation Reports (JCR). The Journal of Allergy and Clinical Immunology was ranked as the next high ranking in eczema with 235 publications and an impact factor of 11.4.

Although the bronchial asthma network is fragmented like in the coupled network, it does not suffer from a tight coupling, and some high-impact journals account for a lot of publications. Among various journals, “J Asthma” ranks first in studying the pathogenic mechanisms, diagnosis, and therapy of childhood bronchial asthma, with 775 publications, and an impact factor of 1.7. “Pediatric Pulmonology” ranked second with 471 published works and an impact factor of 2.7, for the study of health control and prognosis of bronchial asthma (25). Children's bronchial asthma now has a wide international research volume and depth, providing a lot of possibilities in exploring personalized treatment methods.

Journal relationship is more centralized in the studies on allergic rhinitis in children than eczema and bronchial asthma, which, like eczema, “Allergic” is the leading publication in allergic rhinitis (243), focusing on genetic and the emerging therapeutic approach. There is “Pediatric Allergy and Immunology”—the second largest journal on allergic rhinitis, emphasizing the effect of genetic factors on allergic rhinitis in children (26). Hence, it is obvious that genetically influenced factors are a priority trend of new researches to investigate the allergic rhinitis in children.

Research on cough-variant asthma in children is in its early stages but has evolved significantly in recent years. Key journals like “Medicine” and the “Journal of Asthma” have published important studies on the clinical manifestations and treatment of this condition (27). Although there is currently no centralized research network for cough-variant asthma among childhood allergic diseases, this is expected to change as interest and research efforts intensify.

### 3.5 Analysis of citations and co-cited literature

The analysis of highly cited literature identifies key research hotspots, emerging trends, and clinical potential in pediatric allergic diseases. Key studies in eczema research are Tamburini (2016), cited 782 times, and Jakobssen (2014) with 690 citations. Both studies discuss the important factor of microflora, notably gut flora, in the development of allergic diseases, with a particular focus on eczema. Another significant study is Eichenfield's study of specific treatments of childhood eczema with 926 tweets (2014b). This research is the starting point for customizing treatment methods for eczema patients, providing substantial evidence for personalization of treatment. The high number of citations given to these studies is an clear sign of the strengthening and deepening of eczema research in children.

In bronchial asthma research, Sorino (2017), with 1,263 citations and Dharmage (2019), with 679 citations, focus on the field's epidemiology, covering mortality and prevalence. Chung's (2014), with 2,864 citations, stands out, offering significant contributions to advancing conceptual, diagnostic, and treatment guidelines for asthma. Overall, these studies underscore the conceptual framework of bronchial asthma in childhood allergic diseases and have laid a robust ground in advancing personalized treatment options.

In terms of research on pediatric allergic rhinitis, two studies have been particularly influential. Roberts (2018), with 1,754 citations, and Huang (2019d) with 1,409 citations, have made breakthroughs in the field. For instance, Huang demonstrated with comprehensive evidence that allergic rhinitis and atopic dermatitis were linked, reinforcing the interconnected nature of these inflammations within the same immune system. D'amato's study (2015), with 511 citations, has also made significant contributions through the exploration of innovative immunotherapeutic treatments for allergic rhinitis, providing firm evidence in the pursuit of personalized responses.

Regarding pediatric cough variant asthma, Morice (2020)' study with 482 citations emerged as noteworthy due to its attention to the establishment of criteria for the normalization of diagnosis and treatment methods. Sun's study with 19 citations (2019), in contrast, concentrated on the medications necessary to regulate the care of youth suffering from cough variant asthma. These studies' high cites reflect on emerging areas of interest in the research on Childhood cough variant asthma.

**Figure 5:**
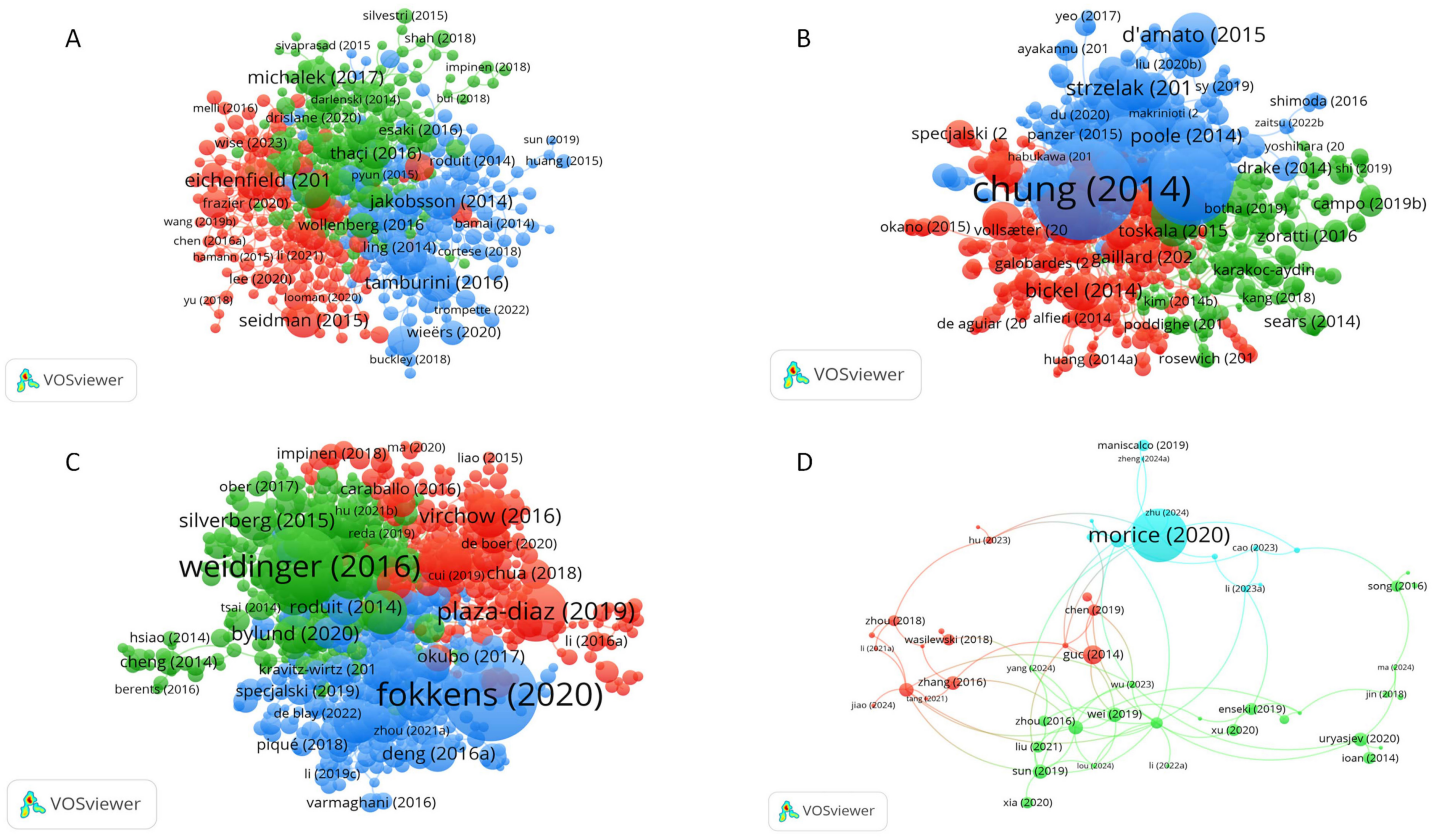
Citation networks of key. **(A)** Citation network for eczema; **(B)** Citation network for bronchial asthma; **(C)** Citation network forallergic rhinitis; **(D)** Citation network for cough-variant asthma. As initially noted, the scope of the study analyzes will be defined by the selection of the search keywords. While it is easy to claim that the selected terms were comprehensive, it is possible that there were niche or still-emergent sub-main-topic areas that may have used these keywords. So, the whole area of research may be underrepresented.

**Figure 6:**
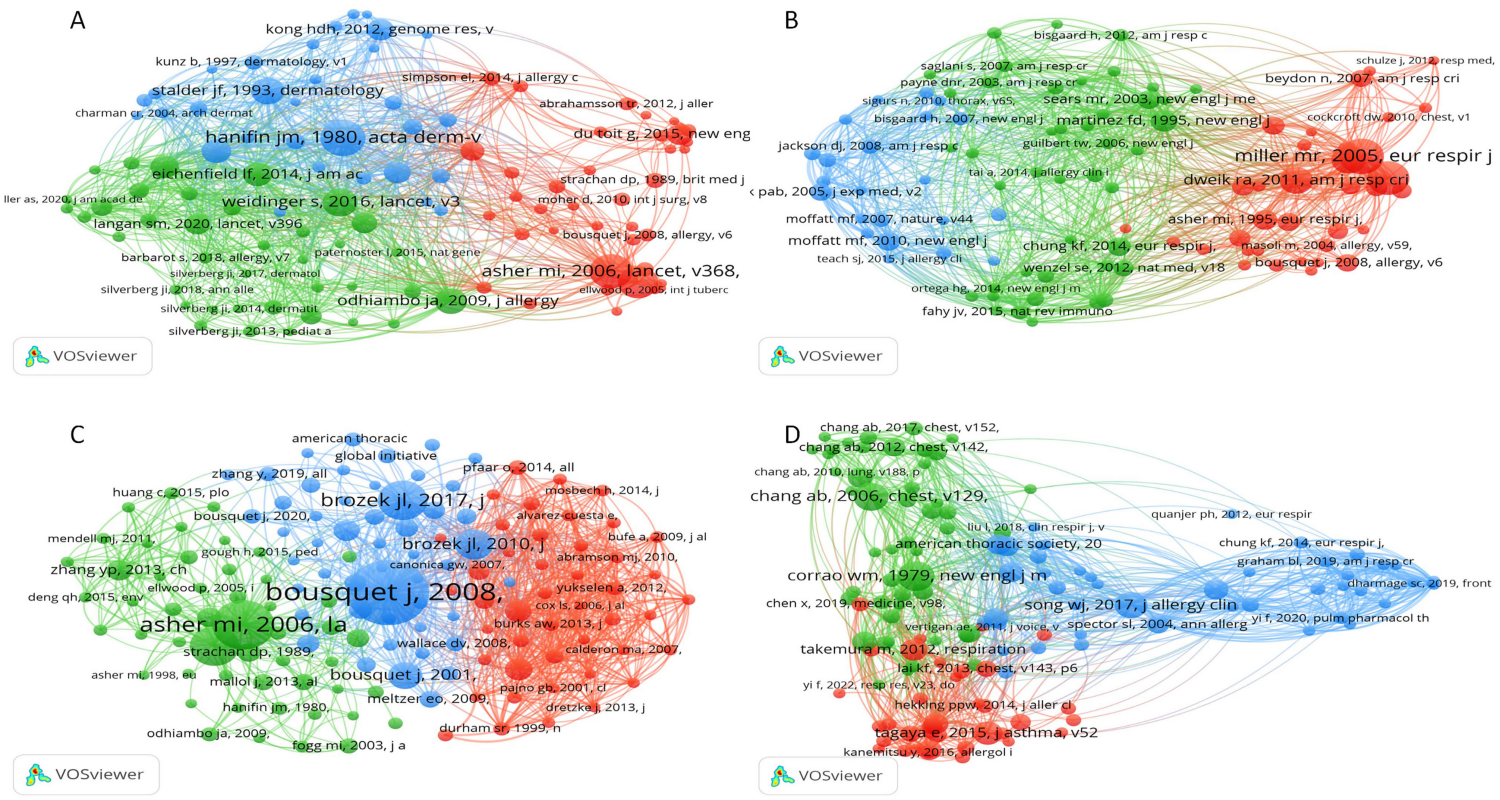
Co-citation analysis of key references. **(A)** Co-citation network in eczema; **(B)** Co-citation network in bronchial asthma; **(C)** Co-citation network in allergic rhinitis; **(D)** Co-citation network in cough-variant asthma. In these networks, each node represents a journal. Node size corresponds to the number of co-citations the journal has received. Lines between nodes indicate that two journals are cited together, with thicker lines denoting a stronger co-citation relationship. Node color represents distinct journal clusters.

Co-citation analysis in eczema research highlights Hanifin JM's significant contributions and broad impact. Asher MI's studies in 1995 and 2006 explored eczema's pathomechanisms and epidemiology, respectively. In bronchial asthma research, spirometry standardization by Miller MR (2005) and Crapo RO (2000) underscores its critical role in diagnosing pediatric bronchial asthma. In allergic rhinitis research, Bousquet J (2008) detailed the link with bronchial asthma, while Asher MI (2006) conducted a comprehensive multinational survey on its prevalence. In cough variant asthma research, Corrao WM (1979) established its link with bronchial asthma, and Chang AB (2006) developed evidence-based management guidelines for children. In conclusion, allergic diseases in children are increasingly a research focus globally, with research depth gradually increasing. This trend indicates a promising future for individualized and refined treatments (Figures 5, 6).

### 3.6 Keyword analysis

**Figure 7:**
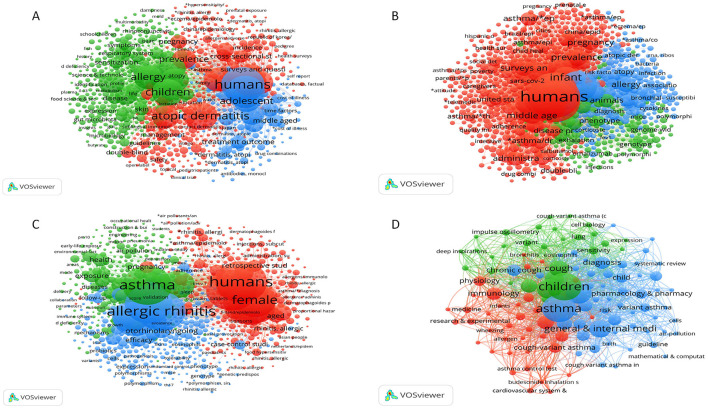
Research hotspots and trends. **(A)** Keyword co-occurrence network of eczema; **(B)** Keyword co-occurrence network of bronchial asthma; **(C)** Keyword co-occurrence network of allergic rhinitis; **(D)** Keyword co-occurrence network of cough-variant asthma. In these networks, each node represents a keyword. Node size reflects the frequency of the keyword's occurrence. Lines between nodes indicate that keywords appear together in the same publication, with thicker lines showing a stronger co-occurrence. Node color indicates the average publication year of articles containing the keyword.

**Figure 8:**
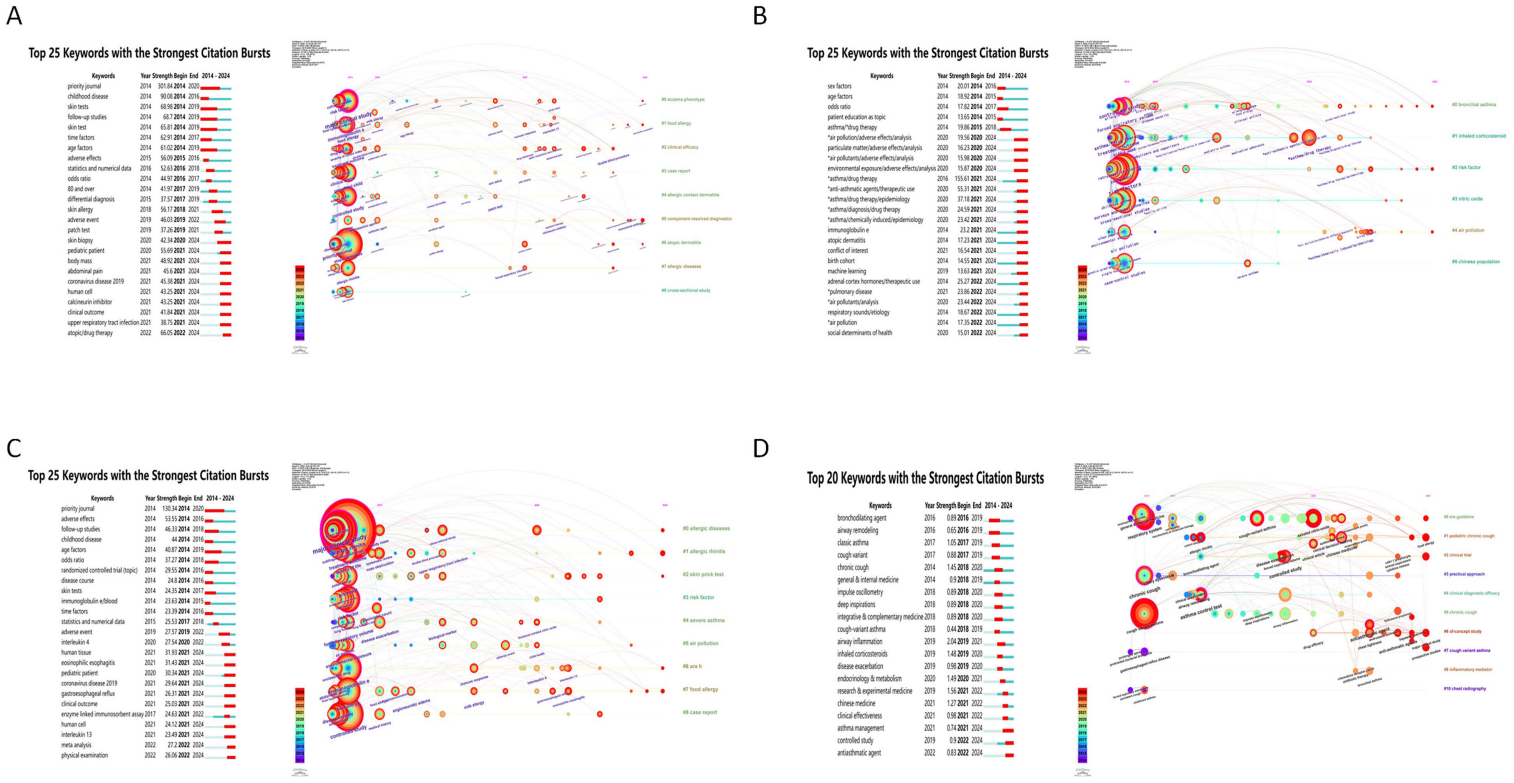
Dynamic trends: top 25 keywords with the strongest citation bursts. **(A)** Top 25 keywords with strongest citation bursts in eczema; **(B)** Top 25 keywords with strongest citation bursts in bronchial asthma; **(C)** Top 25 keywords with strongest citation bursts in allergic rhinitis; **(D)** Top 25 keywords with strongest citation bursts in cough-variant asthma. In these charts, the red bars indicate the time period during which a keyword experienced a significant 'burst' in citations, signaling its emergence as a research frontier. The 'Strength' value quantifies the intensity of this burst.

Bibliometric analyses provide valuable insights into trends and emerging research hotspots in childhood allergic diseases, including eczema, bronchial asthma, allergic rhinitis, and cough variant asthma. Figures 7, 8 show the changing research priorities for each disease over time using co-citation networks and keyword citation bursts. In eczema research (Figure 7A), the top five keywords—“humans,” “child,” “female,” “atopic dermatitis,” and “male”—align with the study topic. Keywords with the strongest citation bursts have been highlighted by the CiteSpace software to identify research frontiers and trends (Figure 8A). On the list, the earliest and most intense outbreak keyword was “priority journal” (2014–2020, intensity 301.84). Additionally, “childhood disease” (2014–2016, intensity 90.08) was the second most intense outbreak keyword. Notably, keywords like “skin biopsy” (2020–2024), “pediatric patient” (2021–2024), “body mass” (2021–2024), “abdominal pain” (2021–2024), “coronavirus disease 2019” (2021–2024), “human cell” (2021–2024), “calcineurin inhibitor” (2021–2024), “clinical outcome” (2021–2024), “upper respiratory tract infection” (2021–2024), and “atopic/drug therapy” (2022–2024) have seen a surge in citations through 2024. This ongoing surge indicates that these research areas have gained significant attention in recent years.

For bronchial asthma (Figure 7B), the five most relevant keywords, “humans,” “child,” “female,” “male,” and “asthma,” are in line with the study's subject. In the keyword burst network (Figure 8B), “sex factors” (2014–2016, intensity 20.01) was at the top, and “asthma/drug therapy” (2021–2024, intensity 155.61) was at the peak. In recent studies, “adrenal cortex hormones/therapeutic use” (2020–2024), “pulmonary disease” (2022–2024), “air pollutants/analysis” (2022–1974), “respiratory sounds/etiology” (2022–2024), “air pollution” (2022–2024), and “social determinants of health” (2022–2024) have been among the keywords. It is a new trend, which means the international concern for the move from demographic epidemiological research into the therapeutic consequences of a given drug and respiratory air pollution causative factors.

In the study of allergic rhinitis (Figure 7C), the keyword choices, “humans,” “child,” “asthma,” “allergic Rhinitis,” and “children” are relevant to the focus. On the keyword outbreak network (Figure 8C), “priority journal” (2014–2020, intensity 130.34) was the first and strongest keyword. The other ones became “adverse effects” (2014–2016, intensity 53.55), a second strong keyword; recent studies have given particular attention to keywords, including “human tissue” (2021–2024), “eosinophilic esophagits” (2021–2024), “pediatric patient” (2021–2024), ” coronavirus disease 2019” (2021–2024), “gastroesophageal reflux” (2021–2024), “clinical outcome” (2021–2024), “human cell” (2021–2024), “interleukin 13” (2021–2024), “meta-analysis” (2022–2024) “physical examination” (2022–2024) “examination” (2022–2024). The continued surge in citations of these keywords indicates that these study areas have attracted much attention recently.

For cough variant asthma (Figure 7D), the top keywords were “children,” “asthma,” “cough variant asthma,” “respiratory system,” and “general &internal medicine,” the subject of the study was in-line with. In the keyword burst network (Figure 8D), “bronchodilating agent” (2016–2019, intensity 0.89) was the oldest keyword and “airway inflammation” (2019–2021, intensity 2.04) was the strongest. There were also recent keywords, such as “asthma management”. The latest keywords were “asthma management” (2021–2024) “controlled study” (2022–2024) and “antiasthmatic agent” (2022–2024)? From the keywords citation surge, we figured out that recent researches had received popular interest on this topic.

## 4 Discussion

### 4.1 Summary of findings

This paper, using bibliometric techniques, examined studies on childhood eczema, allergic rhinitis, bronchial asthma, and cough-variant asthma from three major databases between 2014 and 2024. By mapping publication trends and research hotspots, this analysis provides an objective, quantitative answer to the questions raised in the introduction regarding the current state and future direction of this field. VOSviewer and CiteSpace software were used to visualize the Current Scenario and trends of the research in this area. The analysis revealed that the volume of research varies significantly. of which Eczema is the highest and is still growing. This shows that a sustained concentration of high interest in this area. Persequently, the second largest volume of research was related to bronchial asthma. The study of bronchiosomal asthma is an exciting area, having an extensive history. In recent years, however, there is a slightly declining trend in this field. This fall might not represent less importance but rather the evolution and maturation of the field itself (research transitioned from general epidemiological analysis to more focused, mechanism-based studies—e.g., certain endotypes, biologics, which may not be covered by broad search words, in contrast to increasing recognition of microbiome and newer therapies on eczema, which have been the driving force for its increased publication rates). Research on allergic rhinitis is somewhat small, with unpredictable trends. CVA is the weakest, and its supplies are significantly deficient. The power of research is primarily focused in the United States (most advanced in eczema and asthma) and China (most advanced in rhinitis and CVA). The biggest contributions are made by leading research institutions such as Harvard, the University of California, School of Copenhagen, and Capital Medical University.

In the case of the authors' network we have found that eczema is the most challenging field that highly supports international collaboration (i.e., Silverberg, Ji), asthma network is a most well-defined with a somewhat loxer core team (i.e., Phipatuan, W), Rhinitis has few but very highly-connected teams, and CVA is a highly fragmented collaboration (i.e., Hao, Cl). Co-citation analysis showed core scholars like Silverberg, Bousquet, and Asher as core scholars who formed the foundation of this research field. Eczema and rhinitis research is represented almost only by a few journals (Allergy: pathophysiology and treatment, genetics, rhinitis) while asthma research spreads over a wide array of journals such as J Asthma and a general coverage of topics. The research on CVA is still quite rare and fragmented.

Among the highly-cited literature for each area, milestones are identified for the microbiological research and treatment guideline development, epidemiology and diagnostic conceptualization in asthma, the advances on co-aborbities and immunotherapy in rhinitis, and the development of the diagnostic guideline in CVA. A keyword analysis identifies a shift over the years of focus of research, from epidemiology with a focus on early-stage studies to micro-mechanisms (microbiome, immune pathways, genetics), precision therapies (biologics, JAK inhibitors, AIT), co-morbidities management, environmentally determinism factors (air pollution), emergent factors (COVID-19), etc., and disease-specific studies (e.g., airway inflammation in CVA). However, the maturation level of these areas does not appear to be homogeneous over the fields.

### 4.2 Implications for future clinical research

The bibliometric analysis presented in this paper succinctly follows up on the introduction's description of the need for systematics and points to the knowledge gaps and future research directions for specific high-impact areas.

#### 4.2.1 Deepening the exploration of mechanisms

The popularity of the “microbiome” in eczema research (e.g., Tamburini 2016, Jakobsson 2014; Figure 1), and the significant appearance of the keyword “air pollution” in asthma studies (Figure 8B), denote current research hotspots in these fields. Yet, much of the research is restricted to correlation-level. Likewise, “skin biopsy” in eczema research (Figure 8A) and “allergic rhinitis” (Figure 8C) represent active research areas. In addition, a core group of authors (Silverberg, Phipatanakul, and Ciprandi, Figure 2; Table 2), and active, but fragmented reviewer networks (Figure 2), underlines the importance to explore the cellular and molecular mechanism involved and the heterogeneity of these diseases in depth.

#### 4.2.2 Optimization of treatment studies

“Asthma/drug therapy” (“adrenal cortex hormones/therapeutic use” astrama, Figure 8B), “calcineurin inhibitor” (eczema, Figure 8A), “antiasthmatic agent” (CVA, Figure 8D) are keywords frequently covered in literature. Biological agents (e.g., Thaçi 2016, Simpson 2020b) and highly cited studies on immunotherapies (e.g., D'amato 2015, Calderón 2015b; Durham co-citized, Table 2) further attest the importance of the treatment topic in recent research. This points to a need for further probing of precision and long-term treatment approaches.

#### 4.2.3 Tackling research on co-morbidities

The emergence of the keyword “asthMA” for the context of allergic rhinitis, Figure 7C, combined with the high status and co-citation strength of Bousquet's prominent work (Table 2), as well as Bousquet's typical work (Bousquet 2008, Table 4), points to the interrelated importance of co-morbidivities in the field of asthma/AR. On the other hand, the emergence of the AR keyword “eosinophilic esophagitis” (Figure 8C), along with the presence of indirect allergic processes in the context of research on eczema, also points to the need to address co-morbidities, yet the author network (Figure 2) adjourn distribution (Figure 4) demonstrate that research in each of these areas remains largely separated, revealing an urgent need for integrating research.

#### 4.2.4 Bridging the CVA research gap

Crucially, our analysis quantitatively confirms the significant knowledge gap in cough-variant asthma (CVA) that was a central motivation for this study. The very low number of CVAQ publications (110, Figure 1), the fragmented author network (Figures 2D, 3D), the nonexistent core journal support (Table 3), and keywords still referring to elementary concepts (“airway inflammation,” “asthma management,” “controlled study,” Figure 8D) clearly demonstrate that CVA has been a largely abandoned field in the arena of academic research. It is well established in the scientific literature that highly cited review articles (e.g., Morice 2020; Chang 2006) underscore a significant gap, pointing to the necessity for expanded basic and clinical research efforts.

#### 4.2.5 Explore emerging technologies

The high volume of keywords referring to “meta-analysis” (Figure 8C), and “controlled study” (Figure 8D) denotes an increased need for quality evidence in research. Furthermore, a geographic restriction of geographic distribution of research power in China and United States denotes more international coherence of research. However, it necessitates a focus on more systematic research efforts in order to further expand the global footprint of the research work.

### 4.3 Implications for future clinical practice

The bibliometric analyses published so far have offered important reflections on clinical practice in the realm of pediatric allergic diseases, pointing to identified areas of concern and areas for improvement.

#### 4.3.1 Need to improve diagnostic accuracy

The very low amount of documents on CVP (Figure 1) and fragmented author network (Figures 2D, 3D) clearly hint at substantial clinical underestimation and underdiagnosis of the condition. Consequently, despite the existing guidelines as marked by the high citations of Morice (2020), and the very high co-citations of Chang (Table 2), the low research attention implies an undermining of wide-spectrum of their recommended Application in clinical practice.

#### 4.3.2 Move toward individualized treatment

Recent studies tend to be narrower, focusing on particular drugs (shown in Figure 8B), such as “adrenal cortex hormones/therapeutic usage,” targeted biological agents (Thaçi 2016, Simpson 2020b for eczema), or immunotherapies (such as in D'amato 2015 for AR; Durham co-cited, Table 2), and/or pathways (e.g., “interleukin 13.”) These trends indicate the move toward more specific targeting and, ultimately, toward precision medicine as therapies.

#### 4.3.3 Implement co-morbidity assessment

Keywords for AR, such as “asthma” and “eosinophilic esophagitis” (Figures 7C, 8C), Bousquè's high-co-citation (Table 2) and associated high-c cited literature (Table 4) show that co-morbidity are universal clinical problems that need to be addressed.

#### 4.3.4 Strengthen guidance on environmental factors

Keywords “air pollutution” appear in asthma studies (Figure 8B) and “microbiome” in eczema studies (highly cited literature, Table 4) demonstrate clearly that environment is a high-priority of researches.

#### 4.3.5 Enhance patient self-management

Keywords “amthms management” (Figure 8D) and recurrent research in relation to various therapeutic agents (Figure 8), demonstrate clearly that successful treatment regimens management aspects contribute to the clinical outcomes. Guidelines for standard management requirements have to be given by highly cited guidelines (Table 4).

### 4.4 Strengths of the study

This paper has numerous strengths or, that is, it possesses an advantageous degree of reliability and comprehensiveness. The study adopts a plethora of data sources with three databases of note – Web of Science, Scopus, and PubMed, which ensure the search and the data collection are thorough and comprehensive. The research object is well defined and corresponds with a representative research subject. Four major childhood allergic diseases have been chosen with a well-defined search goal in mind. In adopting objective quantitative analysis, the researcher has employed a bibliometric approach and professional software; thereby minimizing the subjective element and quantitatively uncovering the research landscape. Moreover, visual presentations are embraced in order to convert abstruse, technical data into intuitive maps. Hence, it is easier to comprehend the structure of knowledge and patterns. The research was carried out with the aid of multidimensional analyses, which yield a panoramic view through the time, space, subject and content perspectives. It further acknowledges the frontiers (e.g., microbiomes, biologics, air pollution) and deficiency (e.g., weak CVA studies) of the field, to provide the readers with a valuable source for undertaking the relevant research and practice.

### 4.5 Limitations of the study

Even though the study has its strengths, there are quite a number of limitations associated with it. As initially noted, the scope of the study analyzes will be defined by the selection of the search keywords. While it is easy to claim that the selected terms were comprehensive, it is possible that there were niche or still-emergent sub-main-topic areas that may have used these keywords. So, the whole area of research may be underrepresented. There may be another constraint, and it is connected to language and type restrictions. So basically, this study cannot be the real research for non-English literature or for relatively recent developments when it came to these limitations. Even the best of attempts were undertaken by merging various databases and attempting to overcome the heterogeneity issue associated with them. Institution-and author-mastigging may have been affected as a result of the above-described challenges. It has to be added that bibliometrics is dependent on external characterization. Sometimes the study quality cannot be completely evaluated. There may be other factors like number of citations and so on, that influence the number of citations. The keyword extraction itself and more importantly the clustering operation depend upon citations and the algorithms that execute such operations. This is partially subjective, since any of all possible operations has to be employed. There may also be a kind of time lag between being able to begin with and obtaining results, and when the most recently published studies are included in that scope. Nonetheless, this study is likely to provide a systematic reference that is very valuable with regards to understanding research dynamics in this area.

## Data Availability

The original contributions presented in the study are included in the article/supplementary material, further inquiries can be directed to the corresponding author.

## References

[1] Cowdell F. Knowledge mobilisation: an ethnographic study of the influence of practitioner mindlines on atopic eczema self-management in primary care in the UK. BMJ Open. (2019) 9:e025220. doi: 10.1136/bmjopen-2018-025220 31350238 PMC6661925

[2] Xing P Yu H Li M Xiao X Jiang C Mo L . Characterization of arginine kinase, anovel allergen of *Dermatophagoides farinae* (der f 20). Am J Transl Res. (2015) 7:2815–23. 26885278 PMC4731678

[3] Li X Zhang Y Zhang J Xiao Y Huang J Tian C . Asthma susceptible genes in Chinese population: a meta-analysis. Respir Res. (2010) 11:129. doi: 10.1186/1465-9921-11-129 20868478 PMC2955661

[4] Wei H Li W Jiang Z Xi X Qi G. Clinical efficacy of montelukast sodium combined with budesonide or combined with loratadine in treating children with cough variant asthma and influence on inflammatory factors in the serum. Exp Ther Med. (2019) 18:411–7. doi: 10.3892/etm.2019.7574 31258680 PMC6566116

[5] Kim H-Y Ahn S Yang I-J Park S-Y Kim K. Effect of hataedock treatment on epidermal structure maintenance through intervention in the endocannabinoid system. Evid Based Complement Alternat Med. (2020) 2020:3605153. doi: 10.1155/2020/3605153 32063982 PMC6998750

[6] Novianto E Jacoeb TNA Indriatmi W Suhendro Setiabudy R Setiabudy RD . Effectiveness of cimetidine as adjuvant therapy in the treatment of acute-extrinsic atopic dermatitis: a double-blind randomized controlled trial. Dermatol Ther. (2022) 12:715–26. doi: 10.1007/s13555-022-00688-z 35175572 PMC8940995

[7] Nuttall AGL Velásquez W Beardsmore CS Gaillard EA. Lung clearance index: assessment and utility in children with asthma. Eur Respir Rev. (2019) 28:190046. doi: 10.1183/16000617.0046-2019 31748419 PMC9488960

[8] Xia Q Liu M Li H Tian L Qi J Zhang Y. Network pharmacology strategy to investigate the pharmacological mechanism of HuangQiXiXin decoction on cough variant asthma and evidence-based medicine approach validation. Evid Based Complement Alternat Med. (2020) 2020:3829092. doi: 10.1155/2020/3829092 33178315 PMC7647767

[9] Min K-D Yi S-J Kim H-C Leem J-H Kwon H-J Hong S . Association between exposure to traffic-related air pollution and pediatric allergic diseases based on modeled air pollution concentrations and traffic measures in Seoul, Korea: a comparative analysis. Environ Health. (2020) 19:6. doi: 10.1186/s12940-020-0563-6 31937319 PMC6961284

[10] Hu S Xu S Lu W Si Y Wang Y Du Z . The research on the treatment of primary immunodeficiency diseases by hematopoietic stem cell transplantation: a bibliometric analysis from 2013 to 2022. Medicine. (2023) 102:e33295. doi: 10.1097/MD.0000000000033295 37000105 PMC10063298

[11] Lin F Zhang L Wang Y Fu D Wang Y Zhou X . 20-year bibliometric analysis of fuchs endothelial corneal dystrophy: from 2001 to 2020. BMC Ophthalmol. (2022) 22:255. doi: 10.1186/s12886-022-02468-x 35676652 PMC9175354

[12] He W Yang H Yang X Huang J Wu Z. Global research trends in biological therapy for ankylosing spondylitis: a comprehensive visualization and bibliometric study (2004–2023). Hum Vaccin Immunother. 21:2445900. doi: 10.1080/21645515.2024.2445900 39813123 PMC11740677

[13] Liu Y Sun S Zhang D Li W Duan Z Lu S. Effects of residential environment and lifestyle on atopic eczema among preschool children in Shenzhen, China. Front Public Health. (2022) 10:844832. doi: 10.3389/fpubh.2022.844832 35651861 PMC9149154

[14] Sawane K Nagatake T Hosomi K Hirata S Adachi J Abe Y . Dietary omega-3 fatty acid dampens allergic rhinitis via eosinophilic production of the anti-allergic lipid mediator 15-hydroxyeicosapentaenoic acid in mice. Nutrients. (2019) 11:2868. doi: 10.3390/nu11122868 31766714 PMC6950470

[15] Rajvanshi N Kumar P Goyal JP. Global initiative for asthma guidelines 2024: an update. Indian Pediatr. (2024) 61:781–6. doi: 10.1007/s13312-024-3260-7 39051318

[16] Phipatanakul W Mauger DT Guilbert TW Bacharier LB Durrani S Jackson DJ . Preventing asthma in high risk kids (PARK) with omalizumab: design, rationale, methods, lessons learned and adaptation. Contemp Clin Trials. (2021) 100:106228. doi: 10.1016/j.cct.2020.106228 33242697 PMC7887056

[17] Phipatanakul W Mauger DT Sorkness RL Gaffin JM Holguin F Woodruff PG . Effects of age and disease severity on systemic corticosteroid responses in asthma. Am J Respir Crit Care Med. (2017) 195:1439–48. doi: 10.1164/rccm.201607-1453OC 27967215 PMC5470749

[18] Phipatanakul W. Environmental factors and childhood asthma. Pediatr Ann. (2006) 35:646–56. doi: 10.3928/0090-4481-20060901-08 16999298

[19] Werfel T Allam J-P Biedermann T Eyerich K Gilles S Guttman-Yassky E . Cellular and molecular immunologic mechanisms in patients with atopic dermatitis. J Allergy Clin Immunol. (2016) 138:336–49. doi: 10.1016/j.jaci.2016.06.010 27497276

[20] Asher MI Montefort S Björkstén B Lai CKW Strachan DP Weiland SK . ISAAC Phase Three Study Group. Worldwide time trends in the prevalence of symptoms of asthma, allergic rhinoconjunctivitis, and eczema in childhood: ISAAC phases one and three repeat multicountry cross-sectional surveys. Lancet. (2006) 368:733–43. doi: 10.1016/S0140-6736(06)69283-0 16935684

[21] Li X Brejnrod A Trivedi U Russel J Thorsen J Shah SA . Co-localization of antibiotic resistance genes is widespread in the infant gut microbiome and associates with an immature gut microbial composition. Microbiome. (2024) 12:87. doi: 10.1186/s40168-024-01800-5 38730321 PMC11084089

[22] Creticos PS Gunaydin FE Nolte H Damask C Durham SR. Allergen immunotherapy: the evidence supporting the efficacy and safety of subcutaneous immunotherapy and sublingual forms of immunotherapy for allergic rhinitis/conjunctivitis and asthma. J Allergy Clin Immunol Pract. (2024) 12:1415–27. doi: 10.1016/j.jaip.2024.04.034 38685477

[23] Scala E Madonna S Abeni D Cecchi L Cocuroccia B Dattolo A . A microarray-based IgE-molecular mimicry index (IgE-MMI): a biomarker for disease severity, clinical phenotypes, and therapeutic response in atopic dermatitis? Allergy. (2024) 79:3415–29. doi: 10.1111/all.16377 39495073

[24] Zhao Y Zhang L Wu L Yang B Wang J Li Y . Long-term efficacy and safety of stapokibart for moderate-to-severe atopic dermatitis: 52-week results from a phase 3 trial. Allergy. (2024) 80:1348–57. doi: 10.1111/all.16368 39450683

[25] Lin NY Ramsey RR Miller JL McDowell KM Zhang N Hommel K . Telehealth delivery of adherence and medication management system improves outcomes in inner-city children with asthma. Pediatr Pulmonol. (2020) 55:858–65. doi: 10.1002/ppul.24623 31905264 PMC9125769

[26] Ojwang' V Nwaru BI Hanna-Mari T Heli T Kaila M Ahonen S . Early-pregnancy BMI, maternal gestational weight gain, and asthma and allergic diseases in children. Pediatr Allergy Immunol. (2024) 35:e14240. doi: 10.1111/pai.14240 39282918

[27] Zhou X Zhang Y Liu L Feng X Zhang H. Therapeutic effect of acupuncture combined montelukast sodium on cough variant asthma in children: a protocol for systematic review and meta-analysis. Medicine. (2021) 100:e28048. doi: 10.1097/MD.0000000000028048 34941045 PMC8702241

[28] Eichenfield LF Tom WL Berger TG Krol A Paller AS Schwarzenberger K . Guidelines of care for the management of atopic dermatitis: section 2. Management and treatment of atopic dermatitis with topical therapies. J Am Acad Dermatol. (2014) 71:116–32. doi: 10.1016/j.jaad.2014.03.023 24813302 PMC4326095

[29] Tamburini S Shen N Wu HC Clemente JC. The microbiome in early life: implications for health outcomes. Nat Med. (2016) 22:713–22. doi: 10.1038/nm.4142 27387886

[30] Jakobsson HE Abrahamsson TR Jenmalm MC Harris K Quince C Jernberg C . Decreased gut microbiota diversity, delayed bacteroidetes colonisation and reduced Th1 responses in infants delivered by caesarean section. Gut. (2014) 63:559–66. doi: 10.1136/gutjnl-2012-303249 23926244

[31] Sidbury R Davis DM Cohen DE Cordoro KM Berger TG Bergman JN . Guidelines of care for the management of atopic dermatitis: section 3. Management and treatment with phototherapy and systemic agents. J Am Acad Dermatol. (2014) 71:327–49. doi: 10.1016/j.jaad.2014.03.030 24813298 PMC4410179

[32] Asher I Pearce N. Global burden of asthma among children. Int J Tuberc Lung Dis. (2014) 18:1269–78. doi: 10.5588/ijtld.14.0170 25299857

[33] Fokkens WJ Lund VJ Hopkins C Hellings PW Kern R Reitsma S . European position paper on rhinosinusitis and nasal polyps 2020. Rhinology. (2020) 58:1–464. doi: 10.4193/Rhin20.601 32077450

[34] Weidinger S Novak N. Atopic dermatitis. Lancet. (2016) 387:1109–22. doi: 10.1016/S0140-6736(15)00149-X 26377142

[35] Roberts G Pfaar O Akdis CA Ansotegui IJ Durham SR Gerth van Wijk R . EAACI guidelines on allergen immunotherapy: allergic rhinoconjunctivitis. Allergy. (2018) 73:765–98. doi: 10.1111/all.13317 28940458

[36] Huang K Yang T Xu J Yang L Zhao J Zhang X . Prevalence, risk factors, and management of asthma in China: a national cross-sectional study. Lancet. (2019) 394:407–18. doi: 10.1016/S0140-6736(19)31147-X 31230828

[37] D'Amato G Holgate ST Pawankar R Ledford DK Cecchi L Al-Ahmad M . Meteorological conditions, climate change, new emerging factors, and asthma and related allergic disorders. a statement of the world allergy organization. World Allergy Organ J. (2015) 8:25. doi: 10.1186/s40413-015-0073-0 26207160 PMC4499913

[38] Chung KF Wenzel SE Brozek JL Bush A Castro M Sterk PJ . International ERS/ATS guidelines on definition, evaluation and treatment of severe asthma. Eur Respir J. (2014) 43:343–73. doi: 10.1183/09031936.00202013 24337046

[39] GBD 2015 Chronic Respiratory Disease Collaborators. Global, regional, and national deaths, prevalence, disability-adjusted life years, and years lived with disability for chronic obstructive pulmonary disease and asthma, 1990-2015: a systematic analysis for the global burden of disease study 2015. Lancet Respir Med. (2017) 5:691–706. doi: 10.1016/S2213-2600(17)30336-3 28822787 PMC5573769

[40] Papi A Brightling C Pedersen SE Reddel HK. Asthma. Lancet. (2018) 391:783–800. doi: 10.1016/S0140-6736(17)33311-1 29273246

[41] Fahy JV. Type 2 inflammation in asthma–present in most, absent in many. Nat Rev Immunol. (2015) 15:57–65. doi: 10.1038/nri3786 25534623 PMC4390063

[42] Dharmage SC Perret JL Custovic A. Epidemiology of asthma in children and adults. Front Pediatr. (2019) 7:246. doi: 10.3389/fped.2019.00246 31275909 PMC6591438

[43] Morice AH Millqvist E Bieksiene K Birring SS Dicpinigaitis P Domingo Ribas C . ERS guidelines on the diagnosis and treatment of chronic cough in adults and children. Eur Respir J. (2020) 55:1901136. doi: 10.1183/13993003.01136-2019 31515408 PMC6942543

[44] Usta Guc B Asilsoy S Durmaz C. The assessment and management of chronic cough in children according to the British thoracic society guidelines: descriptive, prospective, clinical trial. Clin Respir J. (2014) 8:330–7. doi: 10.1111/crj.12076 24279754

[45] Sun W Liu H-Y. Montelukast and budesonide for childhood cough variant asthma. J Coll Physicians Surg Pak. (2019) 29:345–8. doi: 10.29271/jcpsp.2019.04.345 30925958

[46] Uryasjev MO. Mихайлович УO, Ponomareva IV, Борисовна ПИ, Bhar MF, Фарес БM, et al. The cough variant asthma. Ter. Ark. (2020) 92:98–101. doi: 10.26442/00403660.2020.03.000404 32598800

