# Supplementary material for: Visualization of childhood allergic diseases based on VOSviewer and CiteSpace
Source: Front Med (Lausanne). 2026 Jan 8;12:1615154. doi: 10.3389/fmed.2025.1615154 (PMC12825032; doi:10.3389/fmed.2025.1615154)
Supplement: Supplementary file 1 [file Table_1.docx]

| Search Strategy | | | |
| --- | --- | --- | --- |
| **Tape** | **WOSCC** | **Scopus** | **Pubmed** |
| Eczema | (TS=(eczema)) OR TS=(atopic dermatitis) AND ((((TS=(children)) OR TS=(infantile)) OR TS=(child)) OR TS=(childhood)) OR TS=(pediatric) | ( TITLE-ABS-KEY ( eczema ) OR TITLE-ABS-KEY ( atopic AND dermatitis ) ) AND ( TITLE-ABS-KEY ( children ) OR TITLE-ABS-KEY ( infantile ) OR TITLE-ABS-KEY ( child ) OR TITLE-ABS-KEY ( childhood ) OR TITLE-ABS-KEY ( pediatric ) ) | (eczema[MeSH Terms]) OR (atopic dermatitis[MeSH Terms]) AND (((children[MeSH Terms]) OR (infant[MeSH Terms])) OR (child[MeSH Terms])) OR (pediatric[MeSH Terms]) |
| Allergic rhinitis | TS=(allergic rhinitis) AND ((((TS=(children)) OR TS=(infantile)) OR TS=(child)) OR TS=(childhood)) OR TS=(pediatric) | TITLE-ABS-KEY ( allergic rhinitis ) AND ( TITLE-ABS-KEY ( children ) OR TITLE-ABS-KEY ( infantile ) OR TITLE-ABS-KEY ( child ) OR TITLE-ABS-KEY ( childhood ) OR TITLE-ABS-KEY ( pediatric ) ) | (allergic rhinitis[MeSH Terms] ) AND (((children[MeSH Terms]) OR (infant[MeSH Terms])) OR (child[MeSH Terms])) OR (pediatric[MeSH Terms]) |
| Bronchial asthma | TS=(bronchial asthma) AND ((((TS=(children)) OR TS=(infantile)) OR TS=(child)) OR TS=(childhood)) OR TS=(pediatric) | TITLE-ABS-KEY ( bronchial AND asthma ) AND ( TITLE-ABS-KEY ( children ) OR TITLE-ABS-KEY ( infantile ) OR TITLE-ABS-KEY ( child ) OR TITLE-ABS-KEY ( childhood ) OR TITLE-ABS-KEY ( pediatric ) ) | (bronchial asthma[MeSH Terms] ) AND (((children[MeSH Terms]) OR (infant[MeSH Terms])) OR (child[MeSH Terms])) OR (pediatric[MeSH Terms]) |
| Cough-variant asthma | (TS=(cough-variant asthma)) OR TS=(cough variant asthma) AND ((((TS=(children)) OR TS=(infantile)) OR TS=(child)) OR TS=(childhood)) OR TS=(pediatric) | ( TITLE-ABS-KEY ( cough-variant AND asthma ) OR TITLE-ABS-KEY ( cough AND variant AND asthma ) ) AND ( TITLE-ABS-KEY ( children ) OR TITLE-ABS-KEY ( infantile ) OR TITLE-ABS-KEY ( child ) OR TITLE-ABS-KEY ( childhood ) OR TITLE-ABS-KEY ( pediatric ) ) | (cough-variant asthma[MeSH Terms]) OR (cough variant asthma[MeSH Terms]) AND (((children[MeSH Terms]) OR (infant[MeSH Terms])) OR (child[MeSH Terms])) OR (pediatric[MeSH Terms]) |
