# Supplementary material for: Visualization of childhood allergic diseases based on VOSviewer and CiteSpace
Source: Front Med (Lausanne). 2026 Jan 8;12:1615154. doi: 10.3389/fmed.2025.1615154 (PMC12825032; doi:10.3389/fmed.2025.1615154)
Supplement: Supplementary file 2 [file Table_2.docx]

**Identification of studies via databases and registers**

Records removed *before screening*:

Duplicate records removed (n = 12230)

Records marked as ineligible by automation tools (n = 0)

Records removed for other reasons (n = 0)

Records identified from*:

Databases (n = 58182)

Registers (n = 0)

**Identification**

Records excluded**

(n = 1729)

Not relevant to the four target allergic diseases (n = 1,100)

Publication type not Article or Review (n = 629)

Records screened

(n = 45952)

Reports sought for retrieval

(n = 44223)

Reports not retrieved

(n = 0)

**Screening**

Reports assessed for eligibility

(n = 44223)

Reports excluded:

Not focused on childhood/pediatric population (n = 180)

Reports of included studies

(n = 44043)

**Included**

*Consider, if feasible to do so, reporting the number of records identified from each database or register searched (rather than the total number across all databases/registers).

**If automation tools were used, indicate how many records were excluded by a human and how many were excluded by automation tools.

Source: Page MJ, et al. BMJ 2021;372:n71. doi: 10.1136/bmj.n71.

This work is licensed under CC BY 4.0. To view a copy of this license, visit <https://creativecommons.org/licenses/by/4.0/>
